# Supplementary material for: Protein kinase C-alpha suppresses autophagy and induces neural tube defects via miR-129-2 in diabetic pregnancy
Source: Nat Commun. 2017 May 5;8:15182. doi: 10.1038/ncomms15182 (PMC5424165; doi:10.1038/ncomms15182)
Supplement: Supplementary Information — Supplementary Figures, Supplementary Tables. [file ncomms15182-s1.pdf]

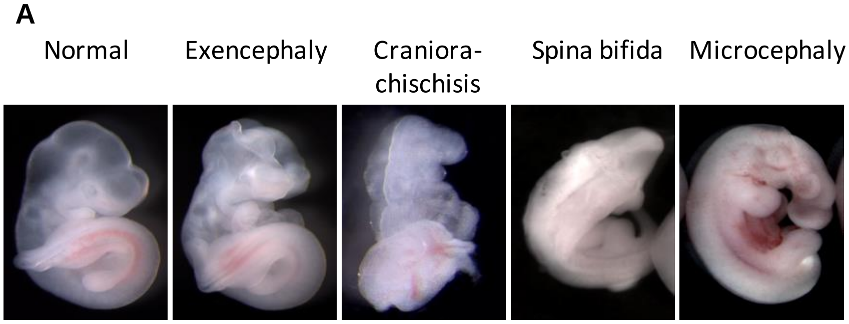

NTD	Number of embryos	% among NTD Embryos
Exencephaly	52	74.3%
Craniorachisc-hisis	6	8.6%
Spina bifida	5	7.1%
Microcephaly	7	10.0%

36 litters; total 280 embryos.

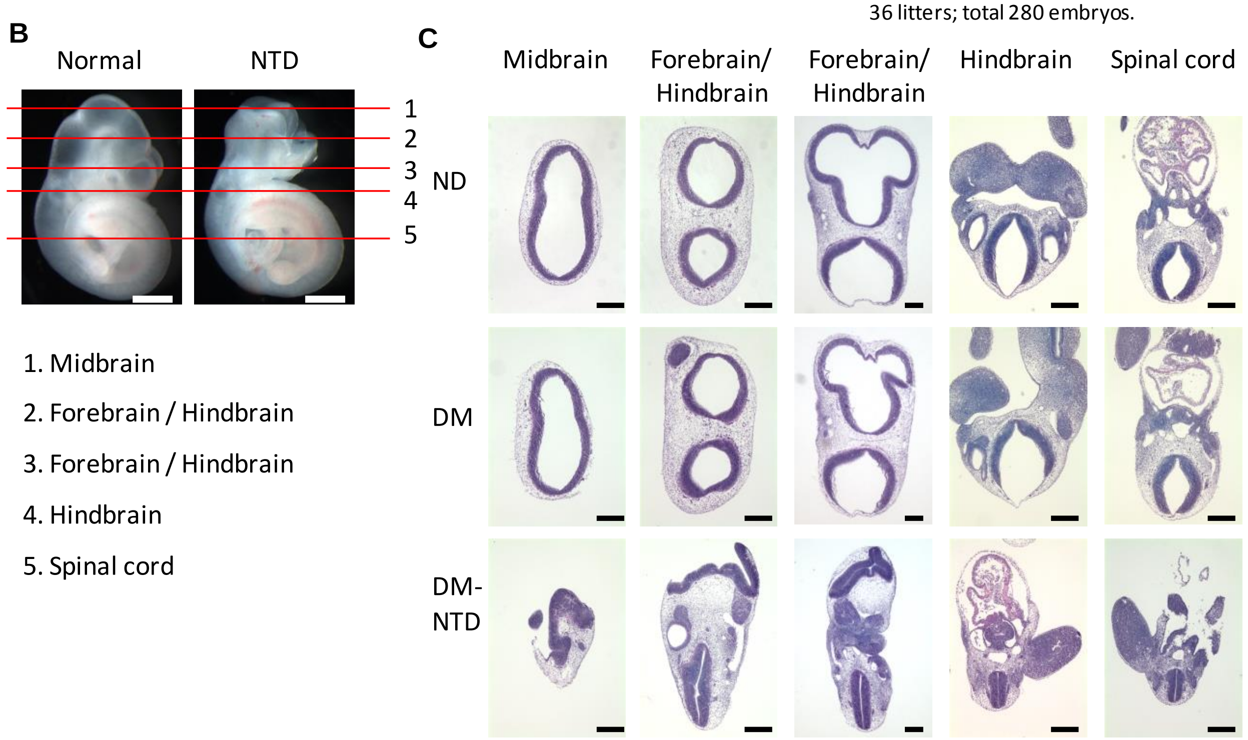

Supplementary Figure 1. Characteristics of NTDs in the mouse model of diabetic embryopathy.
(**A**), types of NTDs in E10.5 embryos exposed to maternal diabetes. (**B**), indicators of histological sectioning plane for **C**. Red lines indicate the levels of sectioning shown below. For each embryo, five sections from top to bottom were chosen to show the morphologic characteristics of the forebrain, midbrain, hindbrain and spinal cord. (**C**), HE staining images of the forebrain, midbrain, hindbrain and spinal cord structures. In **B** and **C**, NTD means exencephaly. Scale bar: 300 µm. ND: nondiabetic dams; DM: diabetic mellitus dams; NTD: neural tube defects.

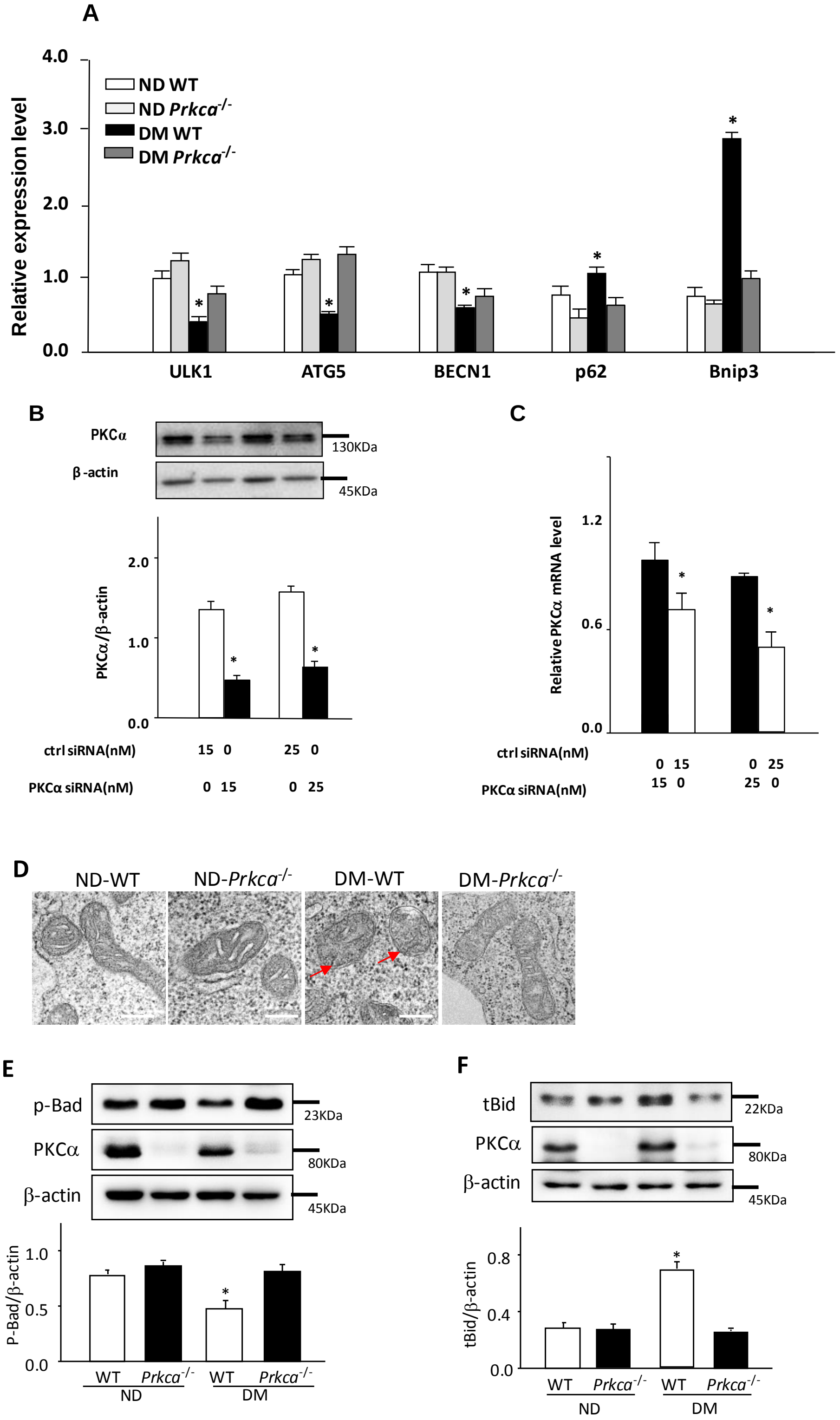

Supplementary Figure 2. *Prkca* gene deletion maternal diabetes-induced autophagy gene aleration and mitochondrial dysfunction. mRNA abundance of ULK1, ATG5, BECN1, p62 and Bnip3 (**A**). The abundance of PKCα protein (**B**) and mRNA (**C**) after cells were transfected with control (ctrl) siRNA or PKCα siRNA at different concentrations. 25 nM PKCα siRNA reduced about 65% endogenous PKCα protein expression and this concentration was chosen for subsequent experiments. Morphology of neuroepithelial cell mitochondria of E8.75 embryos from nondiabetic wild-type (ND-WT), ND-*Prkca*$^{-/-}$, diabetic wild-type (DM-WT) and DM-*Prkca*$^{-/-}$ dams. Normal mitochondria having transversely oriented cristae enclosed by intact outer membranes (**D**). Defective mitochondria with disarrayed or disruptive cristae and decreased electronic density of the matrix () in the DM-WT group. Scale bar: 200 nm. Protein abundance of phospo (p)-Bad (**E**) and tBid (**F**). Experiments were repeated three times using three E8.75 embryos from nondiabetic wild-type (ND-WT), ND-*Prkca*$^{-/-}$, diabetic wild-type(DM-WT) and DM-*Prkca*$^{-/-}$ dams (n = 3) per group. * mean significant difference ($P < 0.05$) compared to other groups.

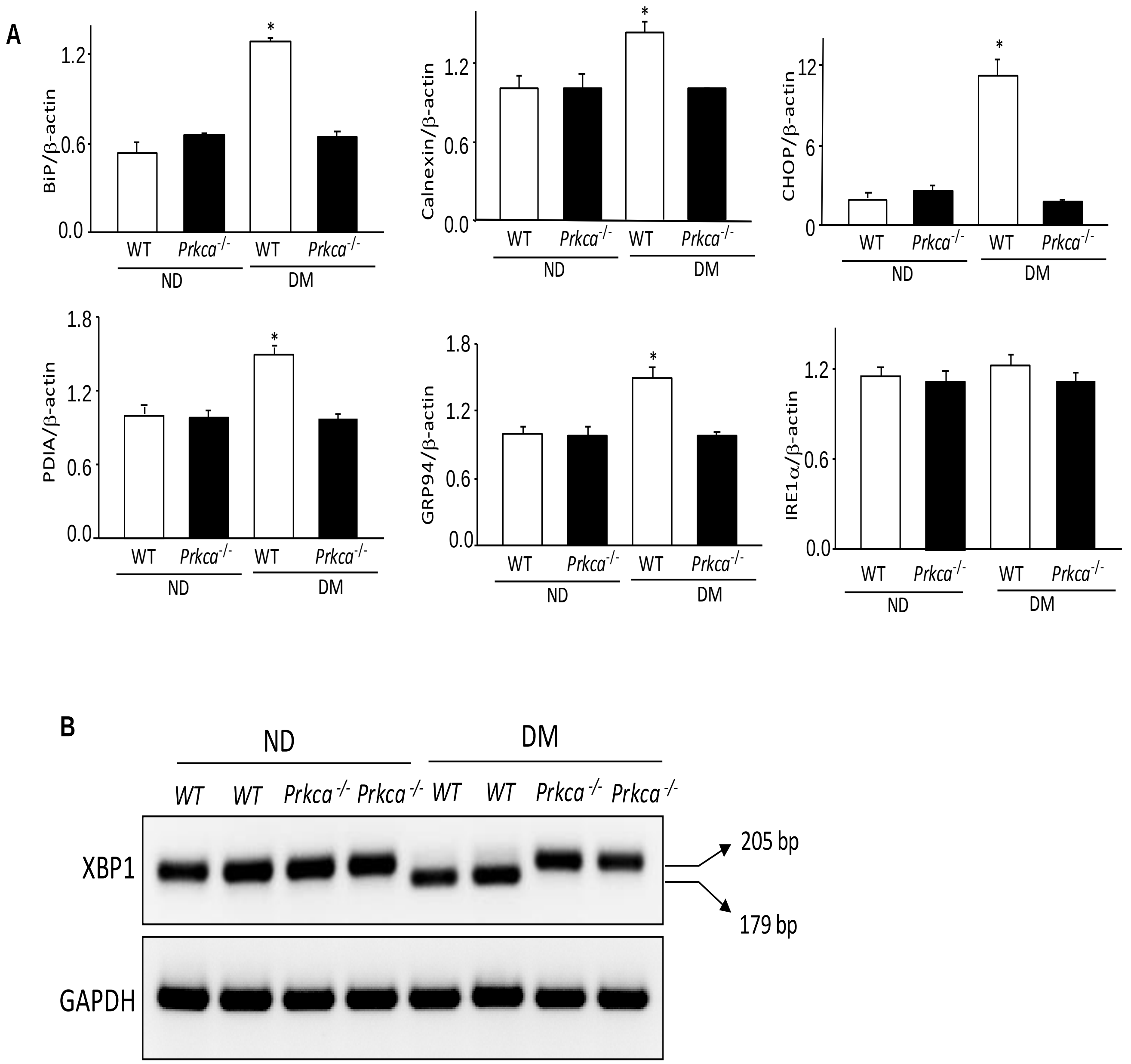

Supplementary Figure 3. *Prkca* gene deletion reverses diabetes-increased ER chaperone gene expression and XBP1 splicing event. A. mRNA abundance of six ER chaperone genes: BiP, Calnexin, CHOP, PDIA, GRP94, IRE1α. Experiments were repeated three times using embryos from three different dams (n = 3) per group. * indicates significant differences (P < 0.05) compared to the other groups. **B.** XBP1 splicing in E8.75 embryos from nondiabetic wild-type (ND-WT), ND-*Prkca*$^{-/-}$, diabetic wild-type(DM-WT) and DM-*Prkca*$^{-/-}$ dams.

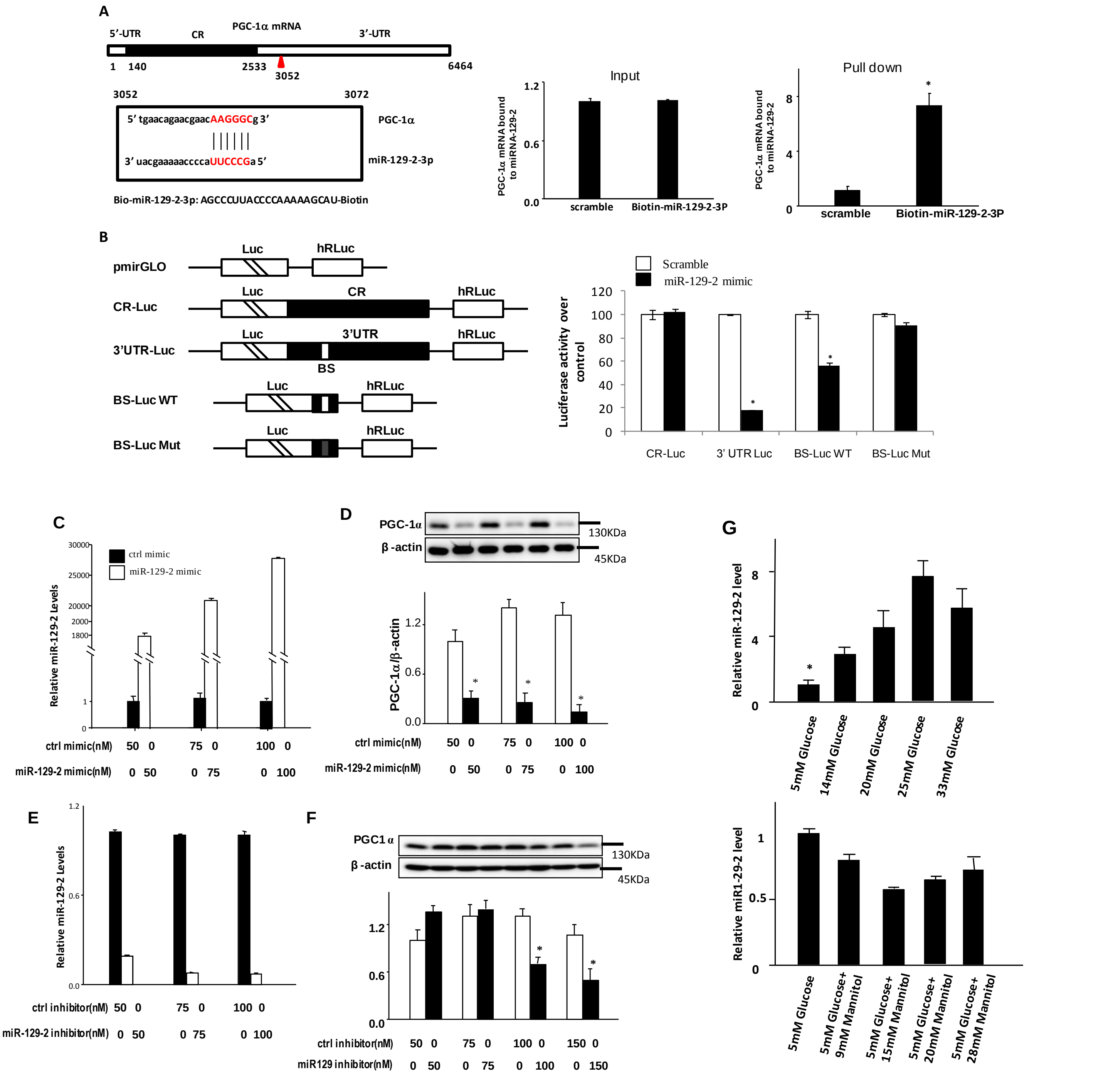

Supplementary Figure 4. miR-129-2 binds to the 3'-UTR of PGC-1α mRNA and represses PGC-1α expression.
A. Schematic representation of the PGC-1α mRNA depicting miR-129-2-3p binding sites in its 3'-UTR. One predicted miR-129-2-3p binding site (position 3052-3072) is located in the 3'-UTR of PGC-1α mRNA. The abundance of PGC-1α mRNA after 48 h biotin-miR-129-2 transfection was shown. **B**. Schematic of plasmids of different chimeric firefly luciferase PGC-1α reporters. Relative luciferase reporter activities driven by the CR (coding region), 3'UTR and BS (a 3'-UTR fragment encompassing the specific miR-129-2 binding site (BS) or having the BS deleted (Mut)) after ectopic overexpression of miR-129-2-3p were shown in the bar graph. Luciferase reporter activities were normalized to the Renilla luciferase activities. Values were the means ± SE from three separate experiments. * indicate significant differences ($P < 0.05$) compared with the scramble group. miR129-2-3p abundance(**C**) and PGC1α protein abundance(**D**) in C17.2 neural stem cells transfected with the control (ctrl) mimic or the miR-129-2 mimic. miR129-2-3p abundance (**E**) and PGC1α protein abundance(**F**) in C17.2 neural stem cells transfected with the control (ctrl) inhibitor or the miR-129-2 inhibitor. (**G**) miR-129-2 levels in C17.2 neural stem cells cultured for 48 hours under normal glucose (5 mM glucose) or high glucose (14, 20, 25 and 33 mM glucose) conditions. High mannitol (9, 15, 20 and 28 mM mannitol) along with 5 mM glucose served osmotic controls of high glucose. Experiments were repeated three times (n = 3) and the quantification of the data were shown in the bar graph. * indicate significant differences ($P < 0.05$) compared with the control groups.

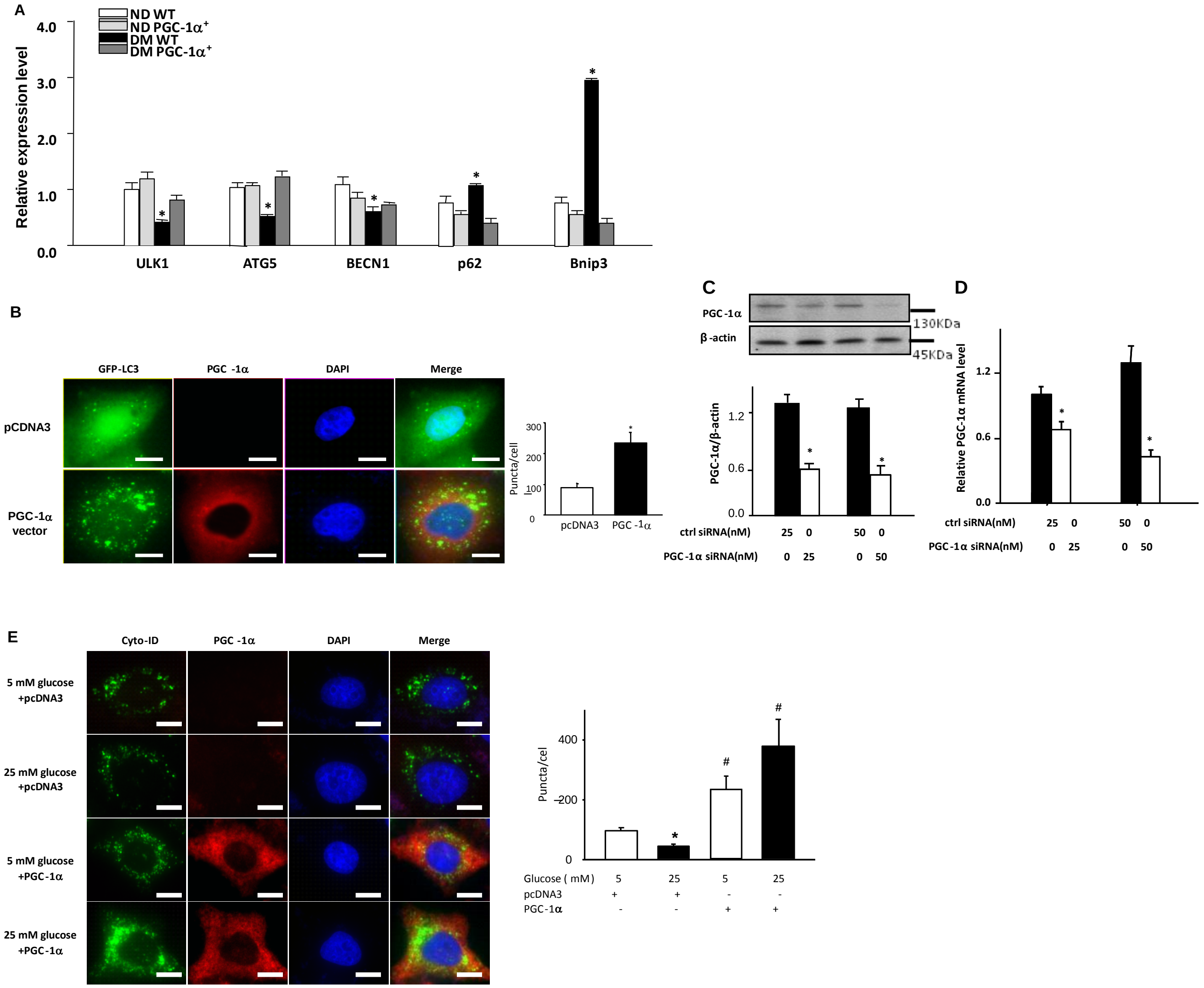

Supplementary Figure 5. ***In vitro PGC-1α*** **overexpression induces autophagosome and restores autophagy suppressed by high glucose.**
A. *PGC-1α* gene overexpression restores the expression levels of maternal diabetes-suppressed autophagy-related gene: mRNA abundance of ULK1, ATG5, BECN1, p62 and Bnip3 in wild-type (WT) and PGC-1α overexpressing (PGC-1α$^+$) embryos. PGC-1α transgenic males mated with nondiabetic (ND) and diabetic (DM) females to generate WT and PGC-1α overexpressing embryos. Experiments were repeated three times using different embryos from three dams (n = 3) per group. * mean significant differences (P < 0.05) compared to other groups. **B.** Autophagosome (GFP punctate) formation was stimulated by PGC-1α tranfections (anti-Flag staining-Red). pcDNA3 blank vector transfections served as controls. Nuclei were counterstained by DAPI. Scale bars:15 µm. The bar graph showed quantification of GFP punctate. **C** and **D**, PGC-1α siRNA effectively silences PGC-1α. PGC-1α protein abundance (**C**) and mRNA abundance (**D**) in cells transfected with the control (ctrl) siRNA or the PGC-1α siRNA at different concentrations. Experiments were repeated three times (n = 3) and quantification of the data were shown in the bar graph. * indicate significant differences (P < 0.05) compared with the control group. **E.** Representative images of Cyto-ID staining puncta, which represented autophagosomes. PGC-1α staining (anti-Flag staining-Red) showed PGC-1α overexpression upon PGC-1α vector transfections. Top row 1 and 2 showed pcDNA3 blank vector transfections as controls. Nuclei were counterstained by DAPI. Scale Bars: 15 µm. The bar graph showed quantification of Cyto-ID staining puncta that represents autophagosome. Five cells per group were quantified and experiments were repeated three times. * indicate significant difference compared with other three groups and # depict significant difference compared with the two 5 mM glucose groups.

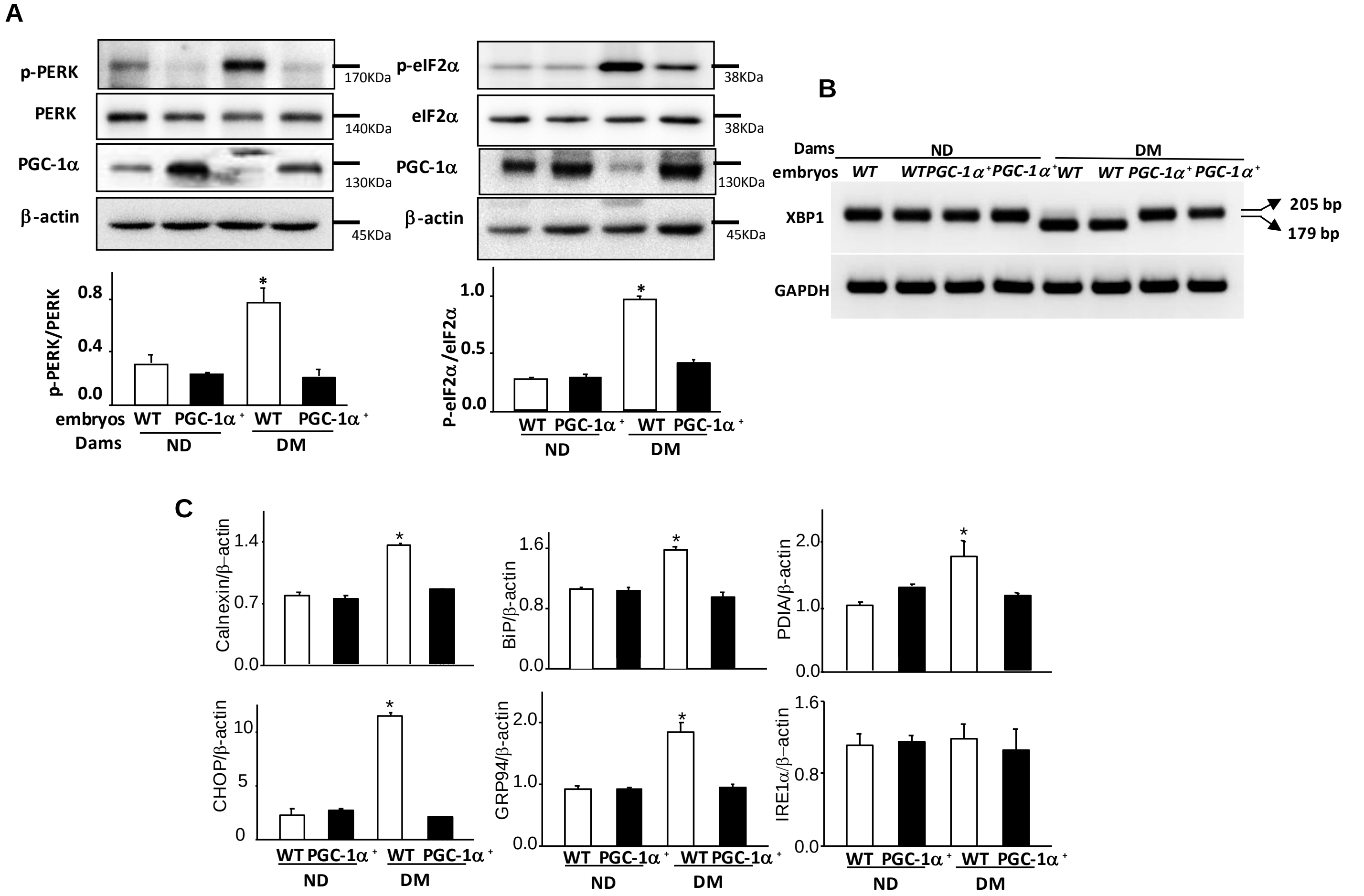

Supplementary Figure 6. ***PGC-1α*** **gene overexpression suppresses maternal diabetes-induced ER stress.**
A. Protein abundance of p-PERK and p-eIF2α. Experiments were repeated three times using embryos from three different dams (n = 3) per group. **B**. XBP1 mRNA splicing was detected in E8.75 embryos by reverse transcription and subsequent PCR. n = 2 means two embryos from separate dams per group. **C.** mRNA abundance of ER chaperone genes: Calnexin, BiP, CHOP, PDIA, GPR94 and IRE1α. Experiments were repeated three times using embryos from three different dams (n = 3) per group. * indicate significant differences ($P < 0.05$) compared to the other three groups.

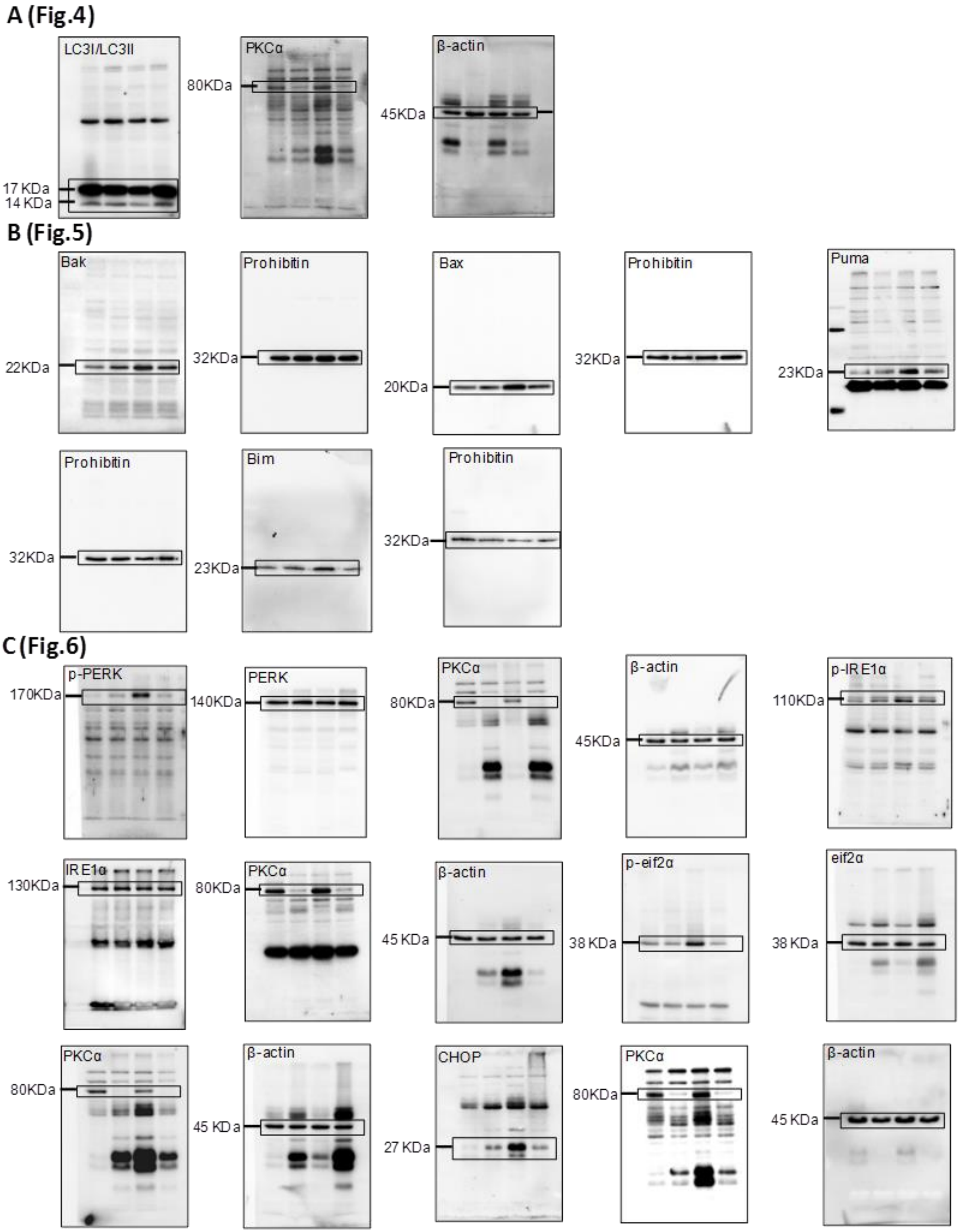

Supplementary Figure 7. Uncropped scans of the most important Western blots

C (Fig.6)

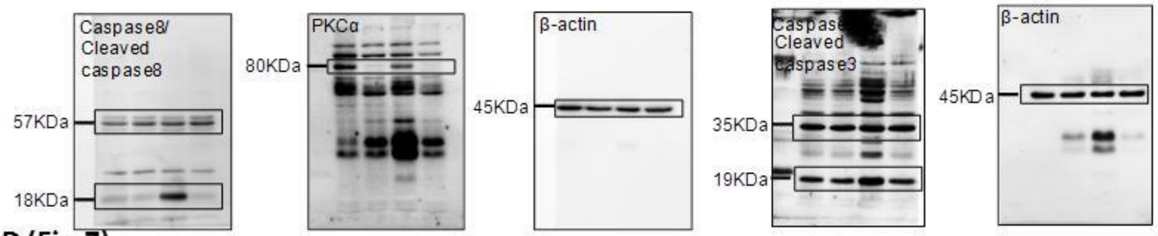

D (Fig.7)

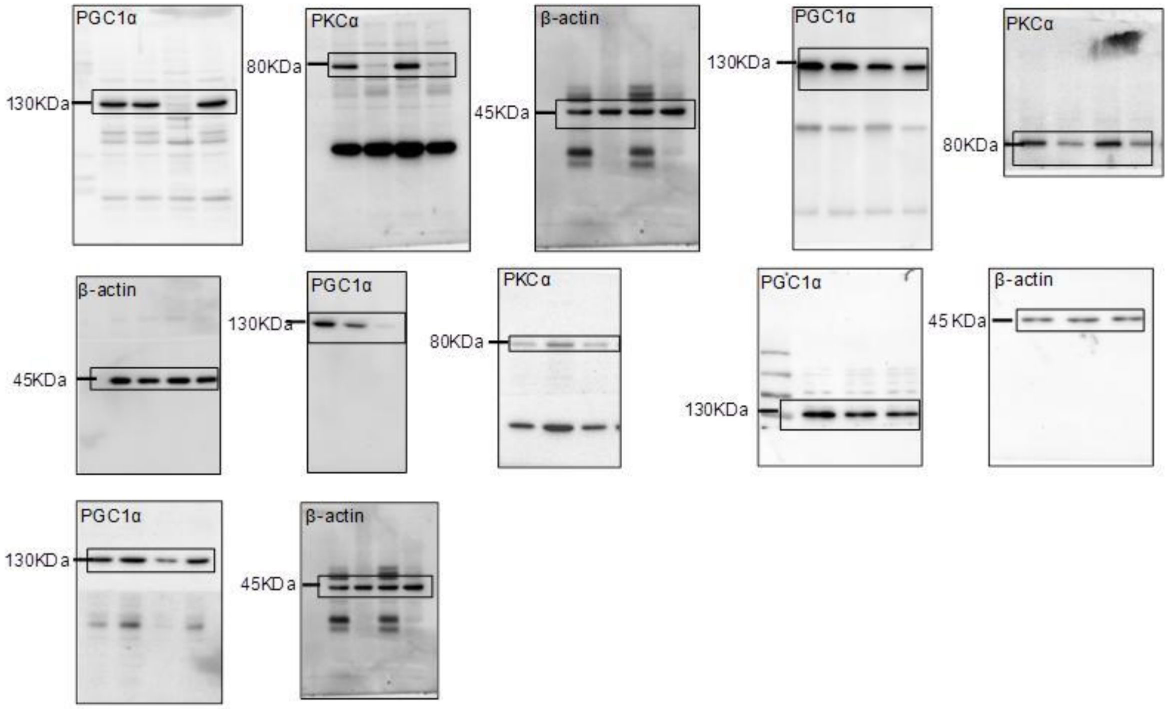

E (Fig.8)

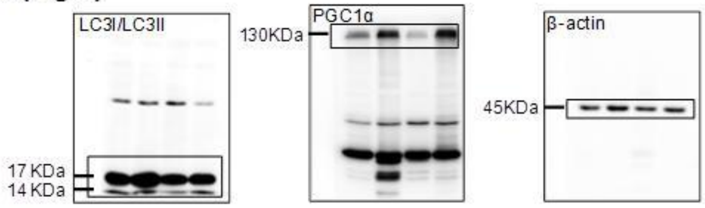

F (Fig.9)

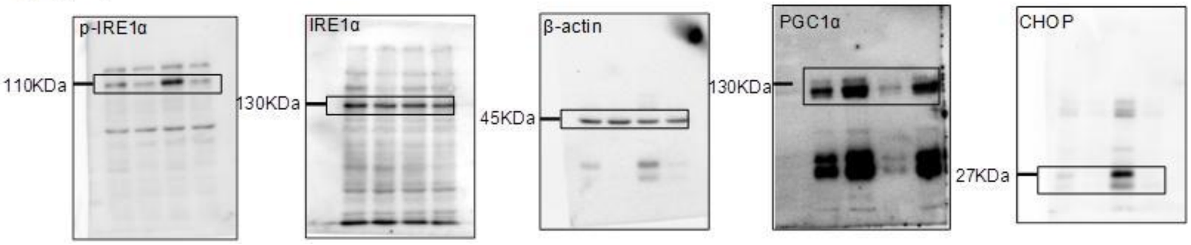

Supplementary Figure 7. Uncropped scans of the most important Western blots

F (Fig.9)

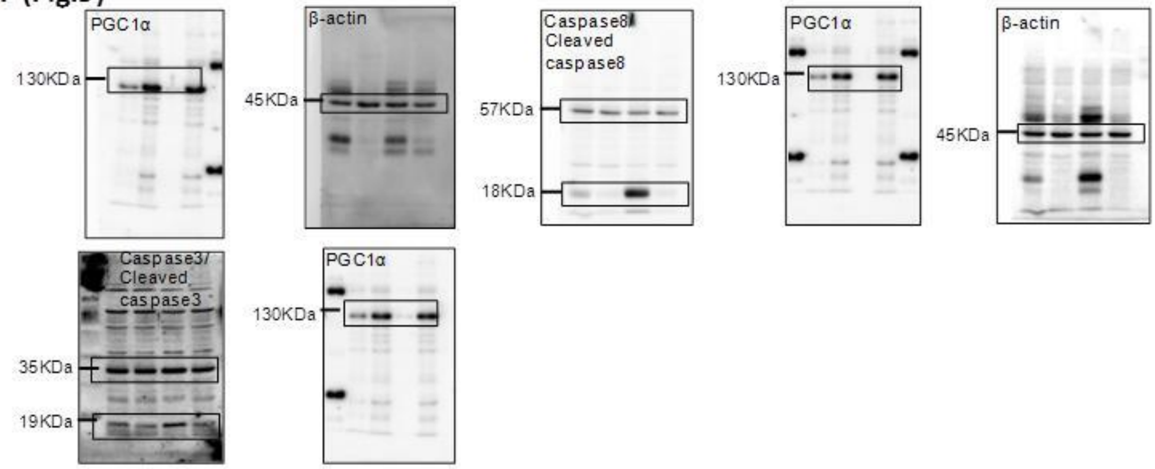

G (Fig.S2)

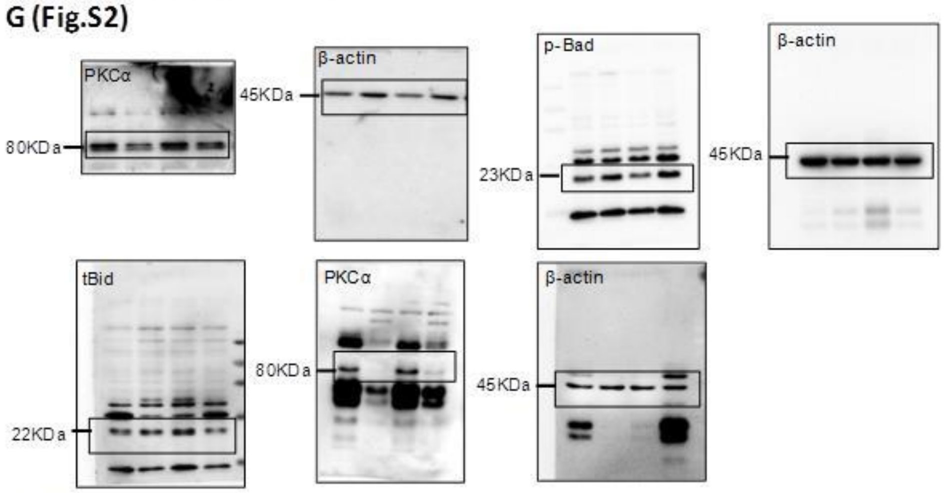

H (Fig.S4)

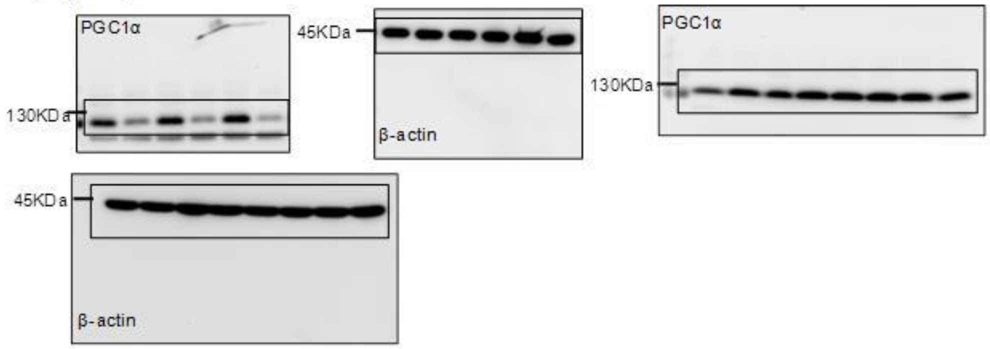

I (Fig.S5)

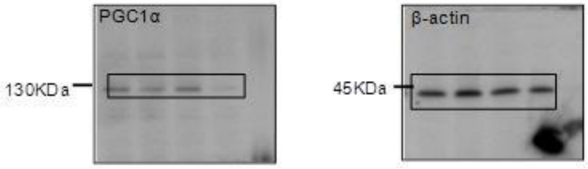

Supplementary Figure 7. Uncropped scans of the most important Western blots

J (Fig.S6)

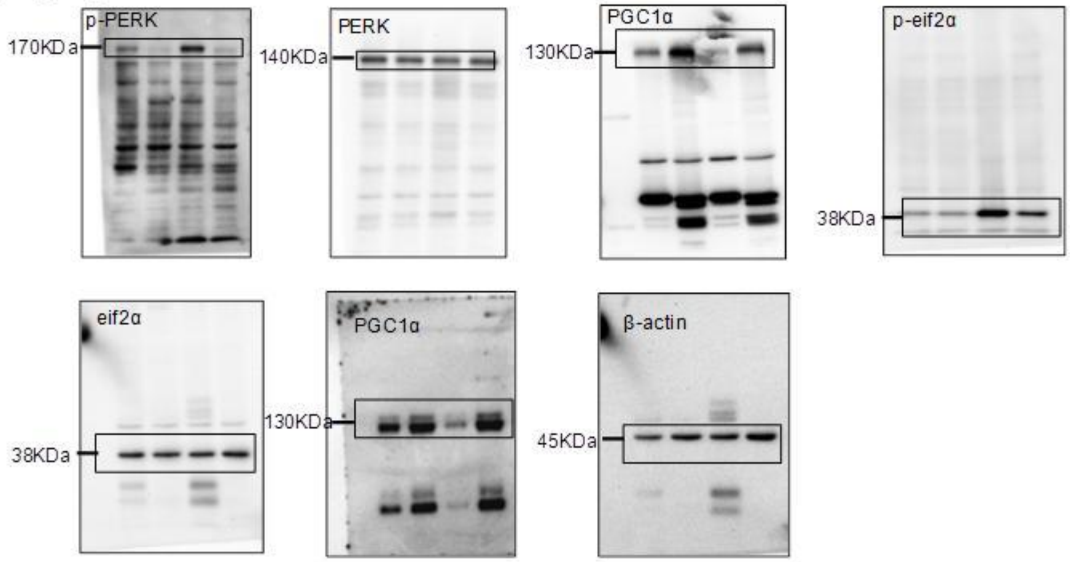

Supplementary Figure 7. Uncropped scans of the most important blots.

Supplementary Table 1 Targeted gene deletion of *Prkca* ameliorates diabetes-induced neural tube defects (NTDs)

Experimental group		Glucose level (mM)	Total embryos	Total defect embryos	NTD rate (%)
ND	WT ♂ x WT ♀ (12 litters)	9.3±0.7	85	0	0
	Prkca$^{-/-}$ ♂ x *Prkca*$^{-/-}$ ♀ (11litters)	9.5±1.1	72	0	0
DM	WT ♂ x WT ♀ (12 litters)	26.1±1.1	78	23*	29.5*
	Prkca$^{-/-}$ ♂ x *Prkca*$^{-/-}$ ♀ (13 litters)	26.1±1.8	88	4	4.5

ND: nondiabetic; DM: diabetic; WT: wild-type; $^{-/-}$: knockout; ♂: male; ♀: female; * indicates significant difference when compared to other groups by using *Chi*-square test.

Supplementary Table 2 PGC-1α overexpression ameliorates diabetes-induced neural tube defects

Experimental group	Glucose level (mM)	Genotype	Total embryos	NTD embryos	NTD rate (%)
PGC-1α ♂ x ND-WT ♀ (13litters)	7.9±1.0	WT	47	0	0
		PGC-1α	45	0	0
PGC-1α ♂ x DM-WT ♀ (13 litters)	24.0±1.5	WT	44	10*	22.7*
		PGC-1α	46	1	2.2

ND: nondiabetic; DM: diabetic; WT: wild-type; ♂: male; ♀: female; * indicates significant difference when compared to other groups by using *Chi*-square test.

Supplementary Table 3 Sequences of primers

Primers name	Primer sources	Primer sequences
GRP94F	Primerbank ID: 6755863a1	TCGTCAGAGCTGATGATGAAGT
GRP94R		GCGTTTAACCCATCCAACTGAAT
CalnexinF	Primerbank ID: 6671664a1	ATGGAAGGGAAGTGGTTACTGT
CalnexinR		GCTTTGTAGGTGACCTTTGGAG
eIF2αF	Primerbank ID: 6857781a1	AGTCCCTGCTCGAATCTTCCT
eIF2αR		TCCCAAGGCAGAACAGATATACC
PDIA3F	Primerbank ID: 6679687a1	CGCCTCCGATGTGTTGGA
PDIA3R		CAGTGCAATCCACCTTTGCTAA
IRE1αF	Primerbank ID: 13249351a1	ACACCGACCACCGTATCTCA
IRE1αR		CTCAGGATAATGGTAGCCATGTC
BiPF	Primerbank ID: 31981722a1	ACTTGGGGACCACCTATTCCT
BiPF		ATCGCCAATCAGACGCTCC
Cox5bF	PrimerBank ID:6753500a1	TTCAAGGTTACTTCGCGGAGT
Cox5bR		CGGGACTAGATTAGGGTCTTCC
SOD2F	PrimerBank ID:31980762a1	CAGACCTGCCTTACGACTATGG
SOD2R		CTCGGTGGCGTTGAGATTGTT
TfamF	primerbank: 1575501a1	ATTCCGAAGTGTTTTTCCAGCA
TfamR		TCTGAAAGTTTTGCATCTGGGT
Nrf1F	primerbank: 13529317a1	TATGGCGGAAGTAATGAAAGACG
Nrf1R		CAACGTAAGCTCTGCCTTGTT
CRF	Own design	AGCTTTTAAAATGGCTTGGGACATGTGCAG
CRR		CTAGGTCGACTTACCTGCGCAAGCTTCTC
3'UTRF	Own design	ATATGCTAGCGGCTGAGGAATGACAGAGAGA
3'UTRR		ACTAGTCGACCTCATGTAACACCGCGTCTG
BS-WTF	Own design	CGATGCTAGCTTGGTGACAGTGTGTGTGCG
BS-WTR		ATATGTCGACACGGTACCGGAGGCTGAC
BS-MutF	Own design	AGCACCGACCCCTTCAAATGGCAGCATTTCC
BS-MutR		GTTCGTTCTGTTCAGGTGCCCCCAAGTCCT
Mmu-miR-129-2 inhibitor	Life technologies: 4464084	Request from Life technologies
miRNA inhibitor Negative Control	Life technologies: 4464076	Request from Life technologies
Mmu-miR-129-2 mimic	Life technologies: 4464066	Request from Life technologies
miRNA Mimic Negative Control	Life technologies: 4464058	Request from Life technologies
Biotin labeled mmu-miR-129-2	miRBase Accession Number: MI0000585	AAGCCCUUACCCCAAAAAGCAU
Biotin labeled negative control	miRBase Accession Number: MI0000038	CGCUCAUUCUGCCGGUUGUUAUG

F: Forward; R: Reverse; CR: coding region for PGC-1α; 3' UTR: 3' UTR for PGC-1α.
